# Comparison of stress distribution around all-on-four implants of different angulations and zygoma implants: a 7-model finite element analysis

**DOI:** 10.1186/s12903-023-03761-x

**Published:** 2024-02-03

**Authors:** Hüseyin Alican Tezerişener, Öznur Özalp, Mehmet Ali Altay, Alper Sindel

**Affiliations:** 1 Private Practice, Antalya, Turkey; 2 Department of Oral and Maxillofacial Surgery, Faculty of Dentistry, Akdeniz University, Campus, Dumlupinar Boulevard, Antalya, 07058 Turkey

**Keywords:** All on four, Atrophic maxillae, Finite element analysis, Stress distribution, Tilted dental implant, Zygomatic implant

## Abstract

**Background:**

In recent years, zygomatic implants and the all-on-four treatment concept have been increasingly preferred for rehabilitation of atrophic maxillae. However, debate continues regarding the optimal configuration and angulation of the implants. The aim of this study was to analyze the biomechanical stress in implants and peri-implant bone in an edentulous maxilla with zygomatic implants and the all-on-four concept, using multiple implant configurations.

**Methods:**

A total of 7 models consisting different combinations of 4-tilted dental implants and zygomatic implants were included in the study. In each model, a total of 200 N perpendicular to the posterior teeth and 50 N with 45° to the lateral tooth were applied. A finite element analysis was performed for determination of stress distribution on implants and peri-implant bone for each model.

**Results:**

Higher stress values were observed in both cortical and trabecular bone around the 45°-tilted posterior implants in all-on-four models when compared to zygomatic implants. In cortical bone, the highest stress was established in an all-on-four model including 45°-tilted posterior implant with 4,346 megapascal (MPa), while the lowest stress was determined in the model including anterior dental implant combined with zygomatic implants with 0.817 MPa. In trabecular bone, the highest stress was determined in an all-on-four model including 30°-tilted posterior implant with 0.872 MPa while the lowest stress was observed in quad-zygoma model with 0.119 MPa. Regarding von Mises values, the highest stress among anterior implants was observed in an all-on-four model including 17° buccally tilted anterior implant with 38.141 MPa, while the lowest was in the including anterior dental implant combined with zygomatic implants with 20,446 MPa. Among posterior implants, the highest von Mises value was observed in the all-on-four model including 30°-tilted posterior implant with 97.002 MPa and the lowest stress was in quad zygoma model with 35.802 MPa.

**Conclusions:**

Within the limits of the present study, the use of zygoma implants may provide benefit in decreasing biomechanical stress around both dental and zygoma implants. Regarding the all-on-four concept, a 17° buccal angulation of anterior implants may not cause a significant stress increase while tilting the posterior implant from 30° to 45° may cause an increase in the stress around these implants.

## Background

Rehabilitation of atrophic maxillae with dental implants has always been a challenging issue due to the certain anatomical and physiological limitations such as severe alveolar bone resorption, pneumatization of maxillary sinus and insufficient subnasal bone volume. To overcome these limitations, researchers have shown a recent interest in less invasive treatment modalities based on full-mouth fixed restorations with minimum number of implants as an alternative to complicated surgical procedures [1–3]. In this regard, zygomatic implants with different numbers and configurations have been used successfully as a viable alternative to advanced surgical procedures or bone grafting in rehabilitation of atrophic maxillae [4–6].

As well as zygomatic implants, a concept called “All-on-Four” has gained much attention as an alternative to augmentation procedures in atrophic maxillae. The All-on-Four concept allows full-arched fixed rehabilitation of edentulous maxillae or mandible on a total of 4 dental implants, with 2 axially placed in anterior region and 2 distally tilted in the posterior region [7–9].

Several publications have appeared in recent years documenting the clinical success of both zygomatic implants and The All-on-Four system in rehabilitation of atrophic maxillae [8, 10, 11]. However, to the authors´ knowledge, no single study exists which compares the biomechanical behaviour of these treatment modalities.

The present finite element analysis (FEA) therefore aimed at investigating the amount and distribution of stress in implants and peri-implant bone in an edentulous maxilla with zygomatic implants vs. All-on-four concept, using different implant configurations.

## Methods

### Finite element model

In the present study, 3-Dimensional (3D) finite element models of maxillae, zygomatic bone, implant fixtures and the superstructure were used to evaluate the amount and distribution of stress in implants and surrounding cortical and trabecular bone. The 3D model of maxillae and zygomatic bone was developed from the computerized tomography (CT) image datasets of a totally edentulous patient with severe maxillary bone resorption (ILUMA, Orthocad, CBCT, 3 M Imtec, Oklahoma, USA). Following the reconstruction of volumetric data with a cross-sectional thickness of 0.2 mm, the sections were exported in Digital Imaging and Communications in Medicine (DICOM) 3.0 format. Bone tissue was separated according to Hounsfield values with interactive segmentation method using 3D-Doctor software (Able Software Corp., MA, USA). and after segmentation, 3D model was obtained with 3D complex render method.

The implants and prosthetic components were scanned with SmartOptics 3D scanner and models were transferred to the software Rhinoceros 4.0 (3670 Woodland Park Ave N, Seattle, WA 98103 USA) in the Standard Tessellation Language (.stl format). Boolean method was used to harmonize the upper and lower parts of the prosthetic components, implant screws and bone tissues in the Rhino software and then force transfer was achieved.

**Fig. 1:**
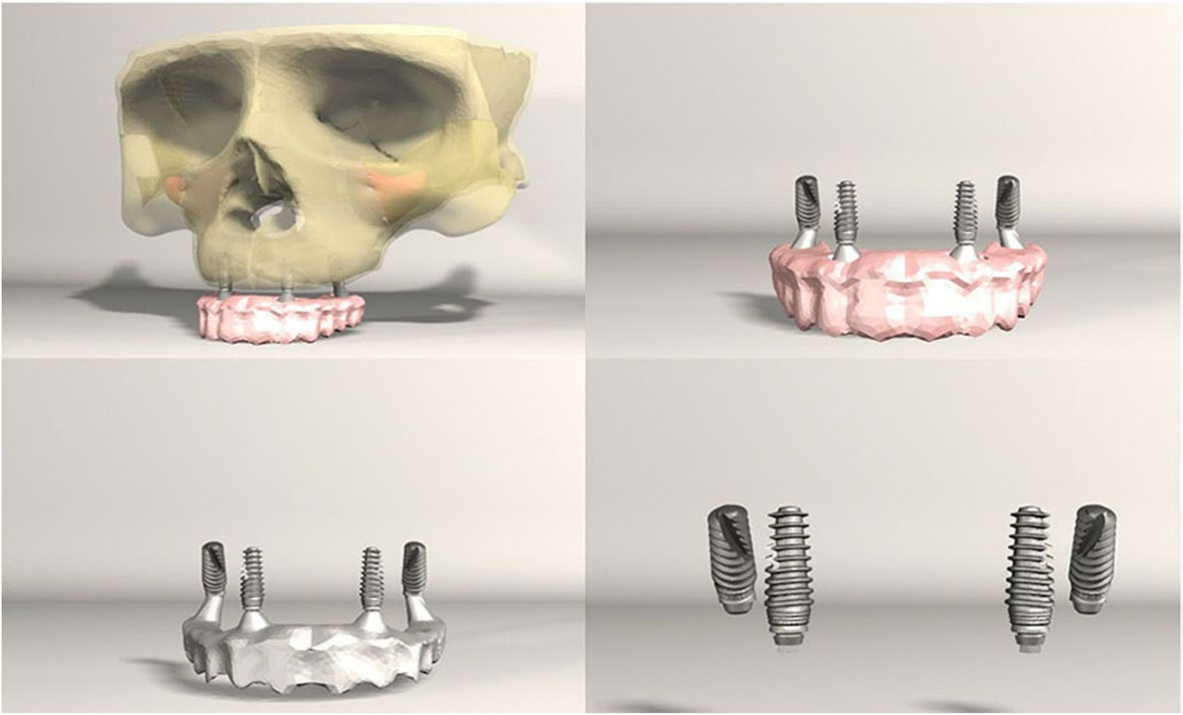
Configuration of implants in Model 1- anterior implants were placed with no angulation and posterior implants were tilted 30° distally

**Fig. 2:**
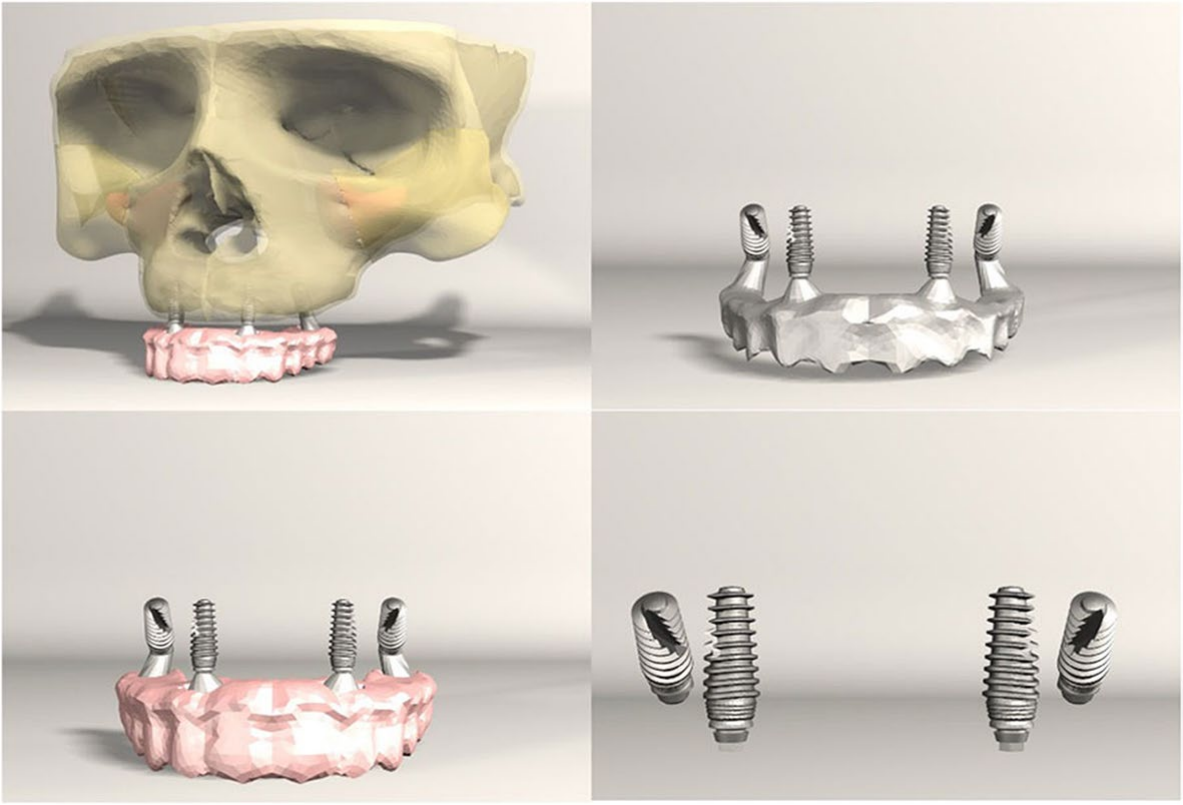
Configuration of implants in Model 1- anterior implants were placed with no angulation and posterior implants were tilted 45° distally

**Fig. 3:**
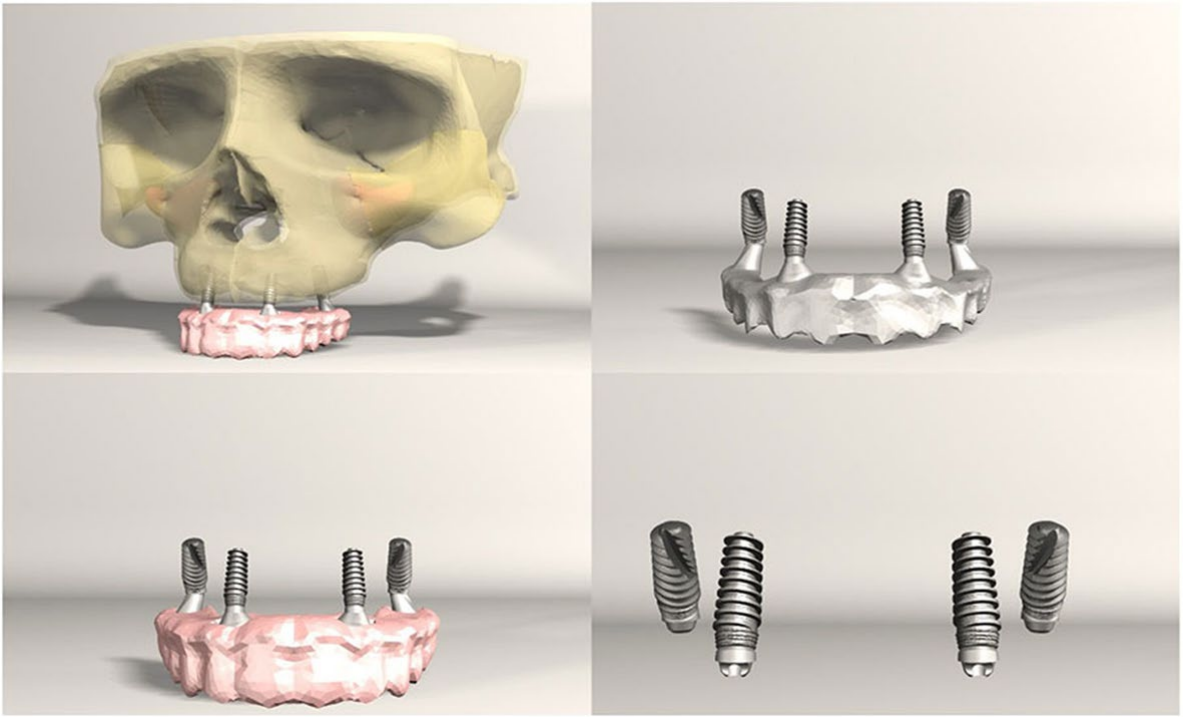
Configuration of implants in Model 3- anterior implants were tilted 17° buccally and posterior implants were tilted 30° distally

**Fig. 4:**
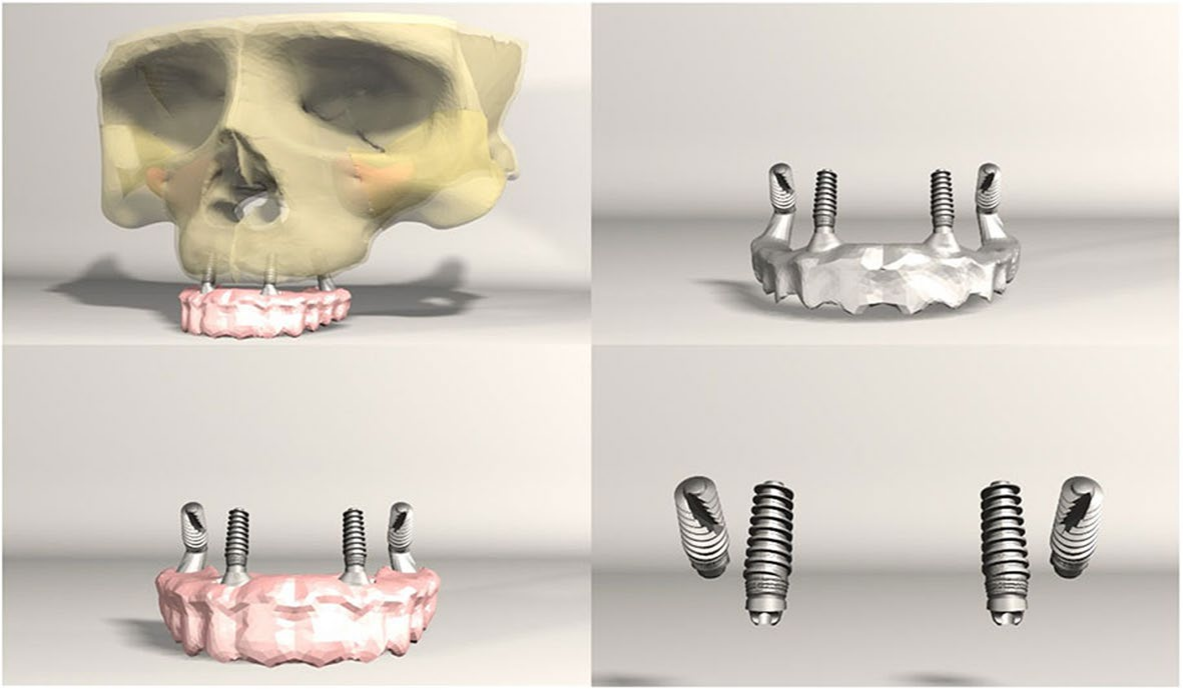
Configuration of implants in Model 4- anterior implants were tilted 17° buccally and posterior implants were tilted 45° distally

**Fig. 5:**
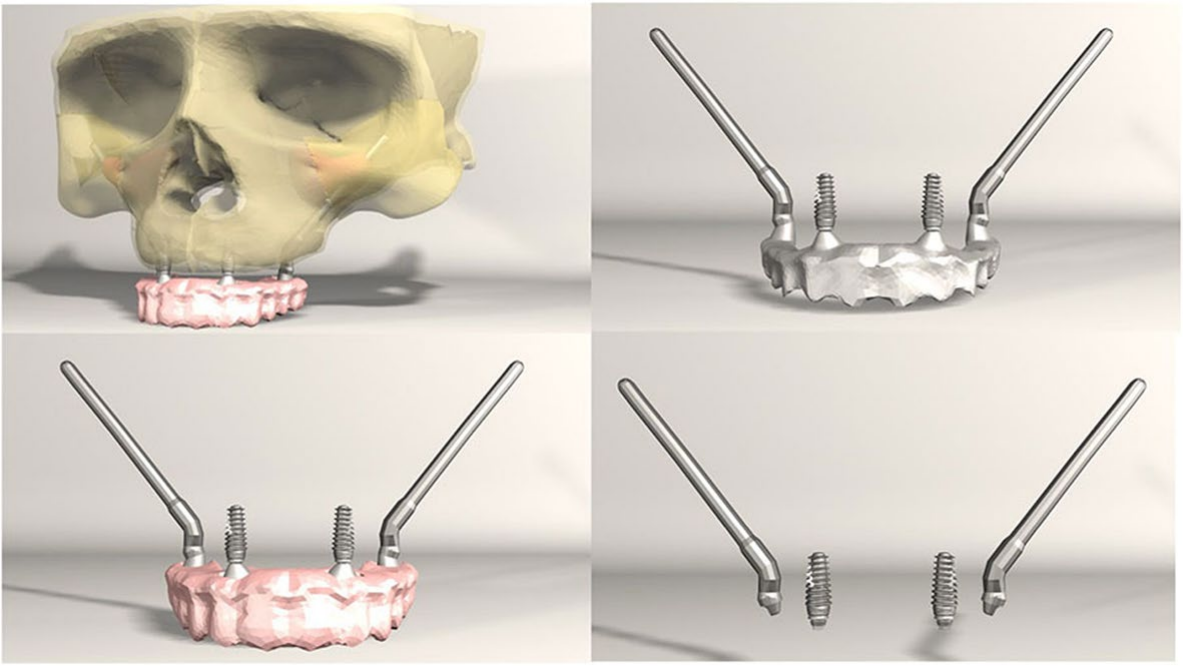
Configuration of implants in Model 5- anterior implants were placed with no angulation and zygomatic implants were placed at a 45° angulation

**Fig. 6:**
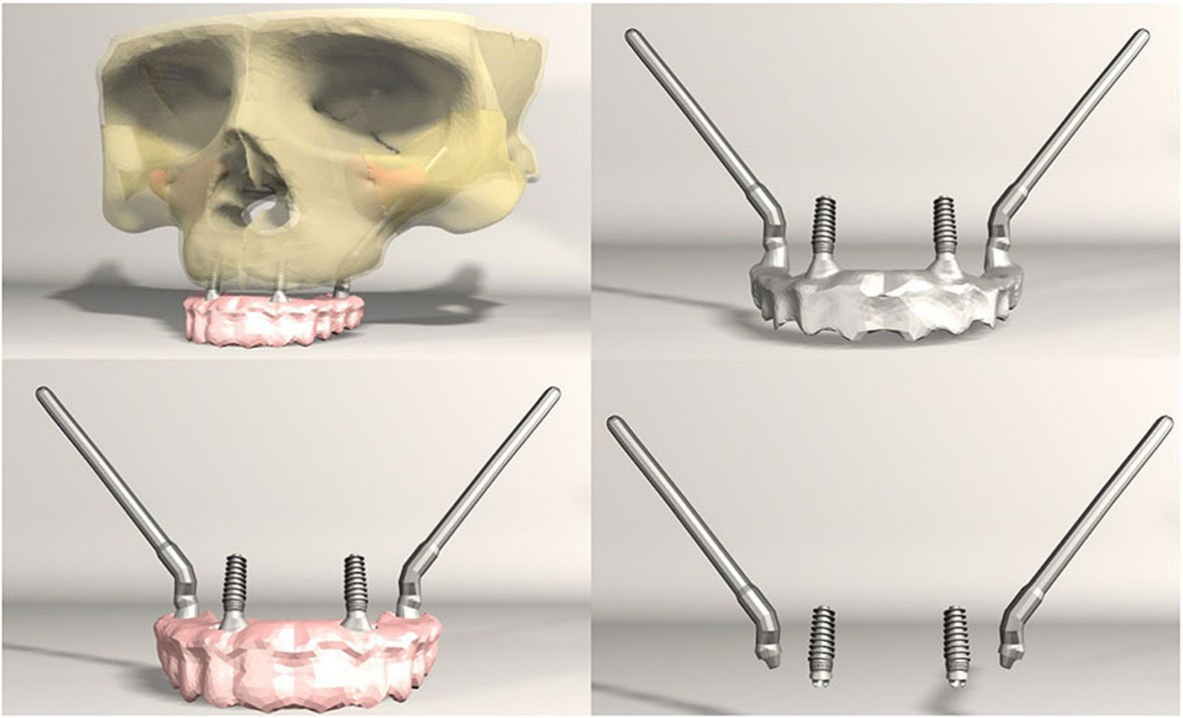
Configuration of implants in Model 6- anterior implants were tilted 17° buccally and zygomatic implants were placed at a 45° angulation

**Fig. 7:**
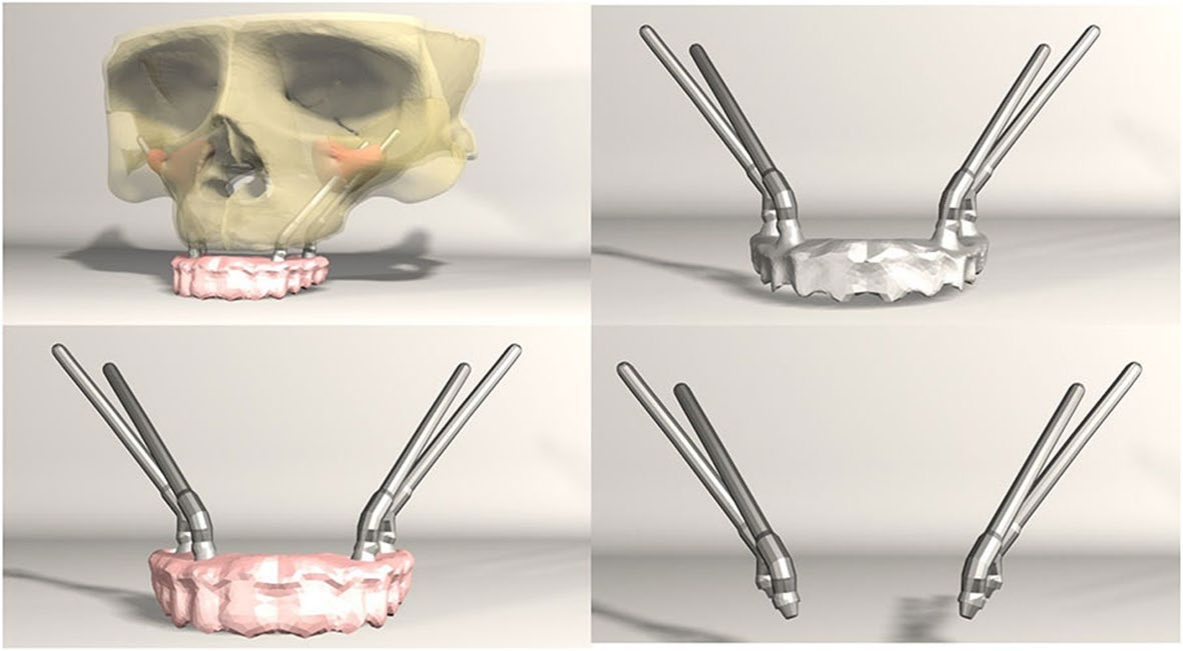
Configuration of implants in Model 7- four zygomatic implants were placed at 45° angulation

FEA was conducted on seven different implant configurations:

- *Model 1:* Bilateral anterior implants (0°) and bilateral posterior implants (30° distally tilted) (Fig. 1).
- *Model 2:* Bilateral anterior implants (0°) and bilateral posterior implants (45° distally tilted) (Fig. 2).
- *Model 3:* Bilateral anterior implants (17° buccally tilted) and bilateral posterior implants (30° distally tilted) (Fig. 3).
- *Model 4:* Bilateral anterior implants (17° buccally tilted) and bilateral posterior implants (45°distally tilted) (Fig. 4).
- *Model 5:* Bilateral anterior implants (0°) and bilateral zygomatic implants (45°) (Fig. 5).
- *Model 6:* Bilateral anterior implants (17° buccally tilted) and bilateral zygomatic implants (45°) (Fig. 6).
- *Model 7:* No anterior implants, bilateral two zygomatic implants (45°) (Fig. 7).

The anterior implants (3.5 × 10 mm) were placed between the lateral incisor and canine while posterior implants (4.3 × 10 mm) were placed between the second premolar and the first molar. The length of the zygomatic implants were determined according to the distance between alveolar crest and the jugal point of the zygomatic bone [12]. The zygomatic implants were positioned so that their coronal ends were along the line of alveolar crest in the premolar and molar areas and apices were embedded in the zygomatic bone. In the 7th model, additional two zygomatic implants were placed as extending from the lateral incisor and canine areas to the zygomatic bone. The implants passed along the lateral wall of the maxillary sinus as described in the sinus slot technique [1].

Cortical bone, trabecular bone, prosthetic units, and implants were moved to the model to reflect their exact morphology. The modeling process was completed by placing the models in the correct coordinates in 3D space using Rhinoceros 4.0 software and VRMesh (VirtualGrid Inc, Bellevue City WA, USA).

Afterwards, the models were transferred to Algor Fempro (ALGOR, Inc. 150 Beta Drive Pittsburgh, PA 15238–2932 USA) in.stl format for analysis.

In the meshing process, the models were created from Brick elements with 10 nodes as much as possible. Elements with fewer nodes were used in the regions close to the center of structures in models. In order to facilitate the analysis process, vertical and narrow regions in the models were made regular by removing linear elements. A mesh convergence test with a tolerance of 5% was applied to ensure mesh size and number of elements. Table 1 presents the number of elements and nodes used for all models.

**Table 1:** Total number of elements and nodes used in models for all models

	**Model 1**	**Model 2**	**Model 3**	**Model 4**	**Model 5**	**Model 6**	**Model 7**
**Number of elements**	304863	315614	332474	333913	239070	279429	159141
**Number of nodes**	65653	69388	75968	75519	52703	65771	42458

All models were assumed to be linearly elastic, homogeneous, and isotropic. The elastic modulus and Poisson ratio values of each structure constituting the models were obtained from the literature and shown in Table 2 [13–15].

**Table 2:** Material properties

	**Young Modulus**	**Poisson’s Ratio**
**Cortical bone**	13700	0.30
**Trabecular bone**	1370	0.30
**Titanium**	110000	0.35
**Sinus**	14000	0.30
**Cr-Co**	218000	0.33
**PMMA**	3000	0.35

**Fig. 8:**
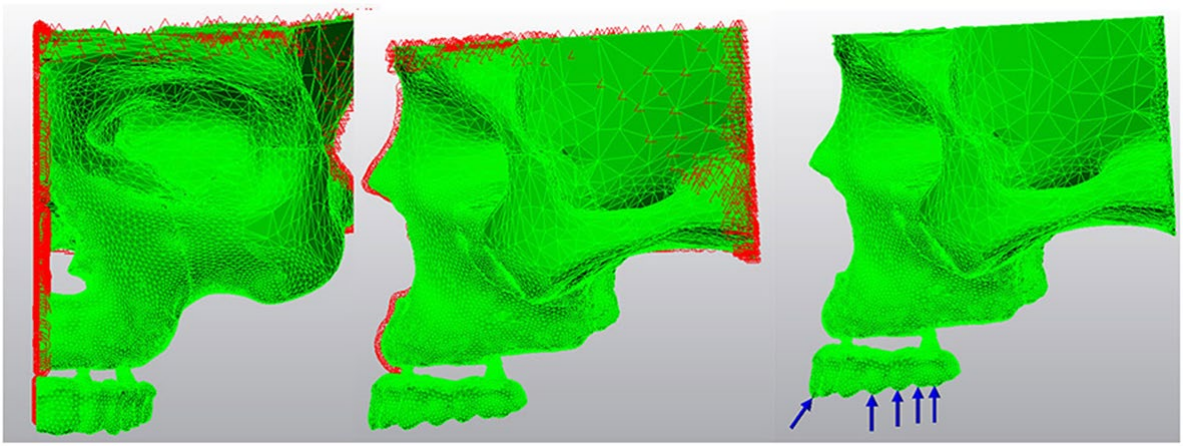
Boundary and loading conditions

The lower and upper parts of the jawbone and the superstructure were fixed to have zero displacement and/or rotation in each degree of freedom (DOF). In each model, the loading zone was selected to mimic the contacts during chewing. To simulate occlusal force, a vertical load of 200 N (N) (50 × 4) was applied on the first molar region and an oblique load of 50 N (45° to the vertical) was applied on the lateral incisor (Fig. 8).

A 3D FEA was run and the von Mises stresses generated on the implants as well as the maximum and minimum principal stress values of the cortical and trabecular bone adjacent to the implants were calculated.

For stress analysis, the von Mises stresses were calculated for dental implants and maximum (tensile) and minimum (compression) principal stresses were calculated for peri-implant cortical and trabecular bone [16]. The highest stress values were quantified by the selection of the node with the maximum value for each structure. The range, color and magnitude scales of the software were used for automatic calculation of stress values. The von Mises, tensile and compression stress values were represented by a color diagram from to red. In the images evaluating the von Mises and tensile stress values, red areas represented high stress regions and colors changed to green and blue as the stress decreased. In the images showing compression stress, blue areas represented high stress regions and as the stress decreased, colors changed to red.

## Results

### Cortical bone

**Fig. 9:**
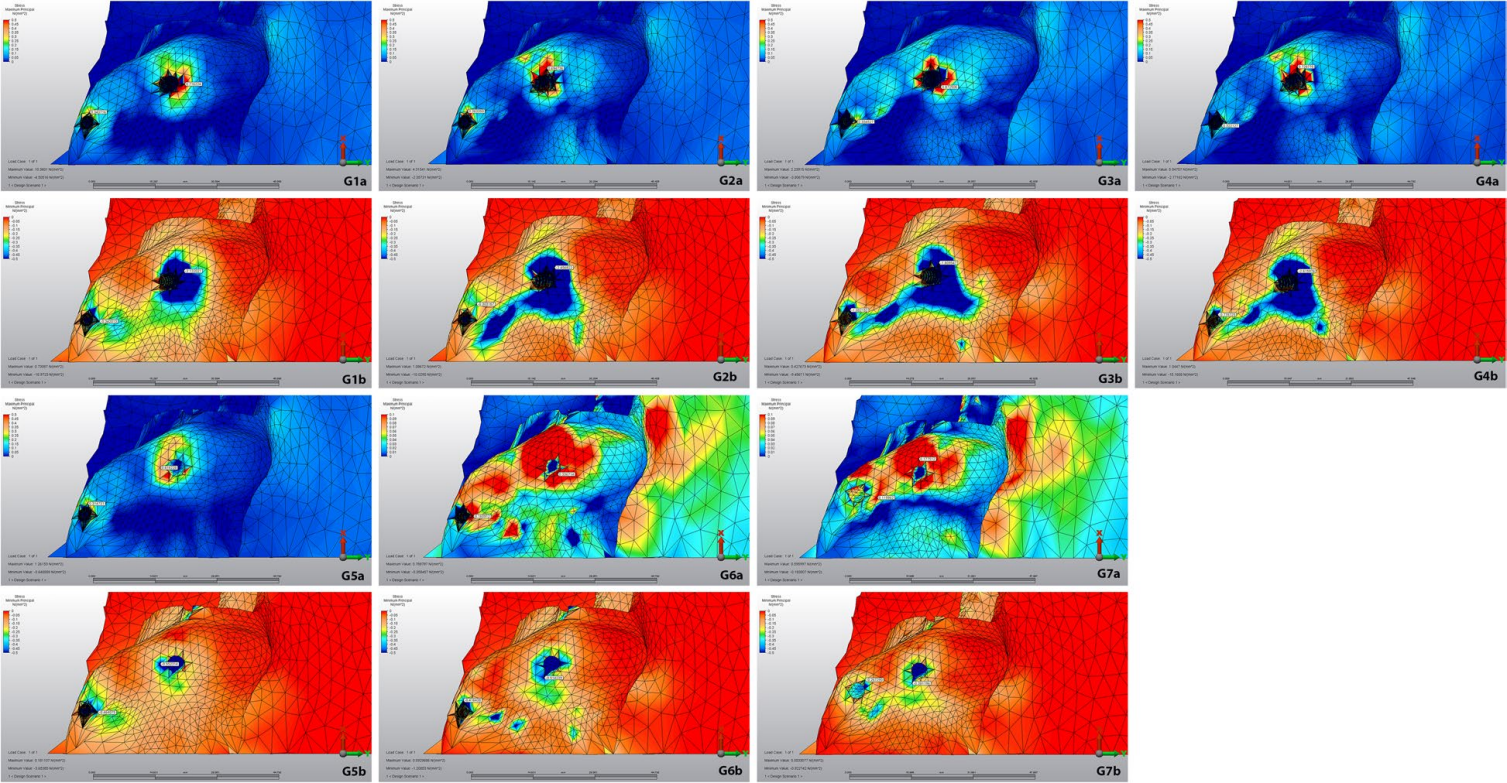
Stress distribution on cortical bone during loading. a: maximal principal stress, red areas were high values, and the blue was low. b: minimum principal stress, blue areas were high values, and the red was low. G1: Group 1, G2: Group 2, G3: Group 3, G4: Group 4, G5: Group 5, G6: Group 6, G7: Group 7

The minimum and maximum principal stress values of cortical bone during loading were shown in Table 3 and Fig. 9. The highest maximum principal stress was established in model 2 with 4,346 megapascal (MPa). The lowest maximum principal stress was determined around the dental implants combined with zygomatic implants in the 5th and 6th models as 0.949 and 0.817 MPa, respectively. The highest minimum principal stress value was found in model 3 with -28.840 MPa. The lowest minimum principal stress was observed with -3,585 MPa around the dental implant in model 6.

**Table 3:** Maximum and minimum principal stress values ​​ in cortical bone

	**Max. Principal Stress**		**Min. Principal Stress**	
	**Anterior Implant**	**Posterior Implant**	**Anterior Implant**	**Posterior Implant**
**Model 1**	2.003	3.514	-15.062	-19.000
**Model 2**	2.901	4.346	-15.214	-27.546
**Model 3**	2.190	4.075	-7.410	-28.840
**Model 4**	2.049	4.332	-5.951	-14.859
**Model 5**	0.949	1.256	-11.766	-14.118
**Model 6**	0.817	3.780	-3.585	-14.463
**Model 7**	1.511	3.291	-7.378	-7.689

### Trabecular bone

**Fig. 10:**
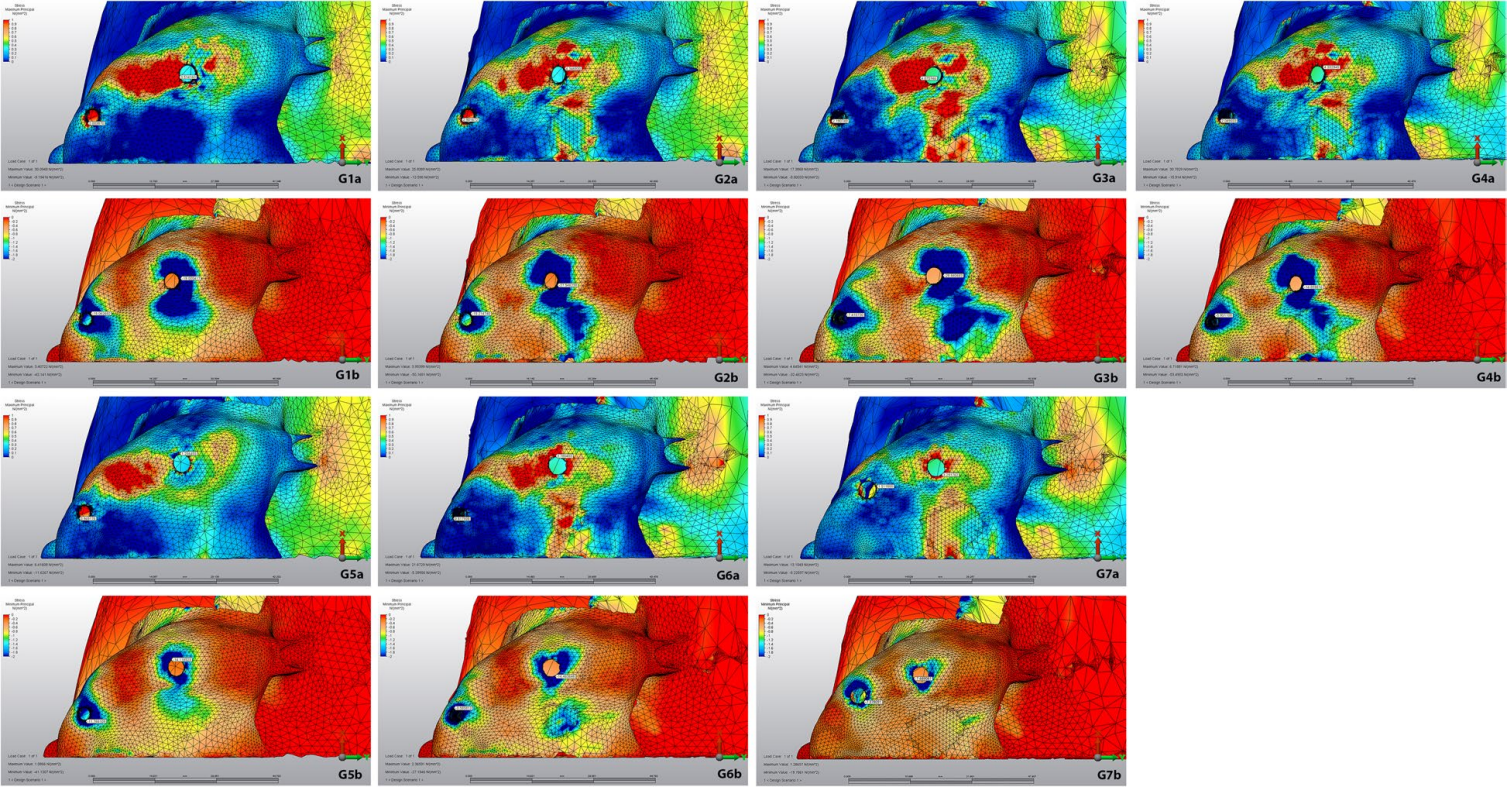
Stress distribution on trabecular bone during loading. a: maximal principal stress, red areas were high values, and the blue was low. b: minimum principal stress, blue areas were high values, and the red was low. G1: Group 1, G2: Group 2, G3: Group 3, G4: Group 4, G5: Group 5, G6: Group 6, G7: Group 7

The minimum and maximum principal stress values of the trabecular bone during loading were shown in Table 4 and Fig. 10. The highest maximum principal stress was determined in model 3 with 0.872 MPa. The lowest maximum principal stresses were observed around the zygomatic implants in model 7 and were calculated as 0.119 and 0.177 MPa. The highest minimum principal stress was determined in model 4 with 2,615 MPa. The lowest minimum principal stress was calculated as 0.267 MPa in model 7 containing 4 zygomatic implants.

**Table 4:** Maximum and minimum principal stress values ​​ on trabecular bone

	**Max. Principal Stress**		**Min. Principal Stress**	
	**Anterior Implant**	**Posterior Implant**	**Anterior Implant**	**Posterior Implant**
**Model 1**	0.343	0.716	-0.342	-2.132
**Model 2**	0.363	0.694	-0.303	-2.456
**Model 3**	0.334	0.872	-1.082	-1.809
**Model 4**	0.322	0.728	-0.778	-2.615
**Model 5**	0.314	0.416	-0.464	-0.552
**Model 6**	0.160	0.336	-0.416	-0.514
**Model 7**	0.119	0.177	-0.267	-0.381

### Implant findings

**Fig. 11:**
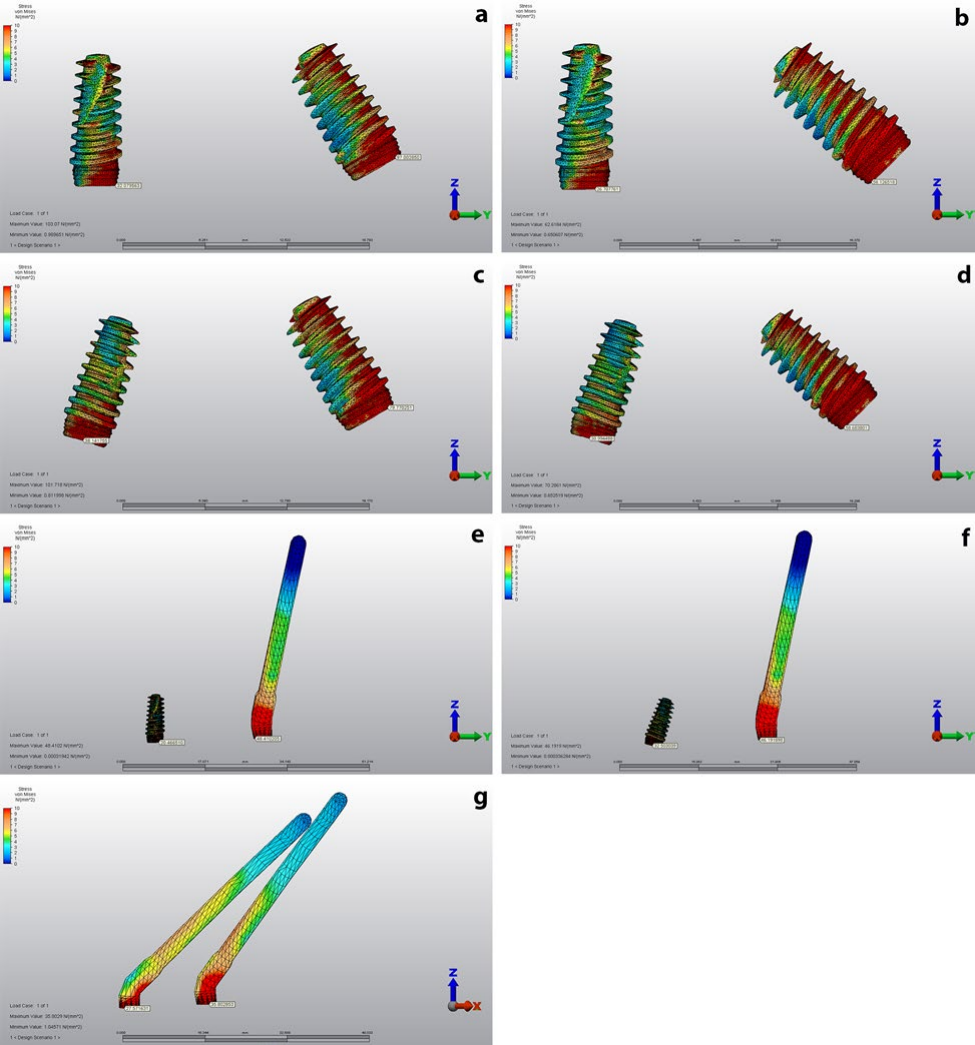
The von Mises stress distribution on implants during loading. Red areas represented the high stress values while the blue areas represented low stress values. a: Group 1, b: Group 2, c: Group 3, d: Group 4, e: Group 5, f: Group 6, g: Group 7

Von Mises values for implants in the anterior and posterior regions during loading were shown in Table 5 and Fig. 11. The highest von Mises value among anterior implants was observed in the 3rd model (38.141 MPa). Among posterior implants, the highest von Mises value was observed in the first model (97.002 MPa). The highest von Misses tension values were observed in the cervical region of the implants. The lowest stress accumulation was detected in the anterior dental implant in model 5 (20,446 MPa). Among posterior implants, the lowest stress accumulation was observed in the 7th model consisting of four zygomatic implants. According to the literature, the tensile value of elastic deformation has been reported to be 1119 MPa titanium implants [17]. In no scenario, the von Misses values in the implants have exceeded the endurance limit of the titanium material.

**Table 5:** Von Misses stress values ​​on implants

	**Anterior Implant**	**Posterior Implant**
**Model 1**	32.079	97.002
**Model 2**	26.707	58.126
**Model 3**	38.141	79.770
**Model 4**	30.994	58.683
**Model 5**	20.466	48.410
**Model 6**	32.503	46.191
**Model 7**	27.571	35.802

## Discussion

In reviewing the literature, no data was found that compared the biomechanical behaviour of zygomatic implants and the All-on-Four system with different angulations of the implants in reconstruction of atrophic maxillae. The results of this study will now be compared to the findings of previous work.

The present study revealed that stress values in cortical bone were higher than in trabecular bone in all models which was in accordance with the findings of other studies investigating stresses on implant and bone tissue [18–20]. One possible explanation for this result may be that the elastic modulus of the cortical bone is higher than the trabecular bone and the cortical bone is the first region to meet the load. In natural dentition, maximum principle stresses are known to stimulate bone formation via periodontal fibers, while minimum principle stresses result in bone resorption [14, 21]. On the other hand, resorption occurs when both minimum and maximum principal stresses exceed certain values due to the absence of periodontium around the implant.

In 2011, Pellizzer et al. examined the distribution of stress in different implant supported crowns and implant planning and reported that the screw retention leads to more stress accumulation [18]. In the same study, they reported an increase in stress accumulation as the angle of implant increased. Similar to the findings of Pellizzer et al., the current study revealed that the highest maximum principal stress in cortical bone was observed in model 2 which suggests that tilting the posterior implant from 30° to 45° caused an increase in tensile stress around this implant compared to the first group. A similar relation was found between the 3rd and 4th models. Regarding trabecular bone, the highest minimum principal stress was found in the 4th model; 45° angulation of the posterior implant resulted in an increase in minimum principal stress around this implant compared to the 3rd model. A similar relation was observed between 1st and 2nd models. Overall, the highest stress values were observed in all-on-four models with 45°-tilted posterior implants.

Although there are many studies in the literature evaluating the success of angled dental implants, few studies have investigated which treatment planning have more successful results when the cantilever length is considered constant [22–24].

In a 5-model finite element analysis study, 4 angled dental implants with angulations of 0°, 15°, 30° and 45° were studied in maxillae models. In the study, they reported that with the increase of implant angulation, the amount of stress in the cortical and trabecular bone decreased and the cantilever length was shortened [23]. Ozan et al. performed a similar finite element analysis study in mandibular models and reported that less stress accumulation occurred by increasing posterior implant angulation and shortening cantilever length [22]. We consider that the reason of the results of our study does not correspond to these finite element analysis studies is that the length of the cantilever was considered constant in the models used in our study and the only determinant variable was the implant angle.

Zygomatic implants combined with 2 to 4 conventional implants has been considered to be the ideal treatment option if the bone volume in anterior maxillae is sufficient [25]. In a 34-month follow-up study, Bedrossian et al. placed 44 zygomatic and 80 premaxillary implants and reported a success rate of 100% in zygomatic implants and 91.25% in conventional implants [25]. However, in a recent meta-analysis, it was concluded that rehabilitating severely resorbed maxillae by using quad zygoma with high prosthetic success and high implant survival rate [26].

There is no general agreement on the ideal angulation of zygomatic implants since the angulation may vary by the anatomy and pattern of the alveolar bone resorption. In 2008, Rossi et al. identified the appropriate sizes, points, and lines for the secure placement of 4 zygomatic implants. In a cadaver study, it has been reported that the ideal angle of zygomatic implants should range between 43.8° and 50.6°, therefore the zygoma implants in the current study were applied with 45° [27].

The lowest maximum principal stress in the cortical bone was determined around the dental implants used with the zygomatic implant in the 5th and 6th models. Similarly, the lowest minimum principal stress in cortical bone was observed around the dental implant in the 6th model. From this point of view, combining dental implants with zygoma implants may help to decrease the stress. On the other hand, in the 7th model with 4 zygomatic implants, the minimum principal stresses in the cortical bone around the anterior and posterior implants were balanced and the total amount of stress was the lowest in this model. This result is in accordance with a study by Varghese et al. which found that the stresses in the quad zygomatic model were lower than in the model with two zygomatic implants combined with conventional anterior implants [28].

The production and destruction balance in the human body increases in the direction of destruction due to aging, and a physiological destruction in the bone structure is observed [29]. Concavity in the anterior region due to the resorption pattern of the maxillae causes difficulties in implant surgery. Considering this resorption pattern, the use of buccally tilted implants and angled abutment in the anterior region is often preferred. In the literature, clinical studies investigating the relation between the use of angled abutments and the success of implant or implant prosthesis show that being straight or angled, or the amount of abutment angle did not affect the success of implants or implant supported prosthesis [20]. In our study, the anterior implants in the 1st and 2nd and 5th models were applied with 0°, while the anterior implants in the 3rd and 4th and 6th models were angled 17° buccally and applied with angled abutments.

In the first 4 models containing only dental implants, the application of the anterior implant with 0° resulted in a reduction in the minimum principal stresses in the trabecular bone around the anterior implants and in the minimum principal stress around the posterior implant according to 3rd and 4th models. In contrast, application of anterior implants with 0° resulted in increased minimum principal stresses in the cortical bone around these implants. In the 5th and 6th models where the anterior dental implant was applied together with the zygoma implant, the application of the dental implant with 0° caused an increase in the minimum principal stress in the cortical bone around the anterior implant, while decreasing the maximum principal stress around the posterior implant.

When the anterior and posterior stresses were compared, higher stresses were obtained in the posterior region in all models. The same was true when comparing the implants in the anterior and posterior regions. The angled implants placed in the posterior region had higher stress values than those in the anterior region.

The present study has several limitations due to the nature of finite element models. First, 100% osseointegration between implants and the surrounding bone was assumed. In clinical situations, the percentage of osseointegration could be reduced by various factors such as inflammation, medications, and metabolic diseases. Another situation that needs to be known is related to the interpretation of images in finite element analysis. In finite element analysis, high-value red areas represent the permanent deformation of the material. However, this is not valid for soft or hard vital tissues, but for solid models. According to the Frost's theory, the results of this study can be interpretated as the regions with the highest stress value are the regions exposed earliest to resorption [30]. However, there is absolutely no conclusion that resorption will occur in the areas where the highest stress occurs. As well, in the current study, several simplifications were performed including the assumption that cortical bone and trabecular bone were homogenous and isotropic, whereas, in a clinical scenario, bone anisotropy is a well-known significant factor that affects stress and strain in peri-implant bone [31]. Furthermore, it has been previously reported that loading values above the bearing capacity of bone will initiate resorption [30]. In the present study, none of the loadings applied to the models exceeded the bearing limit of the cortical and trabecular bone. However, this finding should also be interpretated with caution because it is also known that continuous occlusal loads may cause high stress at the same point in the alveolar bone which may result with bone resorption [32].

Overall, above-mentioned limitations were valid for all models evaluated in the current study, since the main purpose of it was to compare the stresses in different implant configurations rather than stating exact values.

## Conclusions

Within the limitations of this study, the following conclusions were drawn:

1. In all-on-four models, tilting the posterior implants from 30° to 45° increased the stress in cortical and trabecular bone around the posterior implants. Applying anterior implants with 17° angulation buccally did not cause a significant increase in stress values when compared to 0°.
2. Combining the zygoma implants with dental implants may be beneficial in decreasing biomechanical stress around both dental and zygoma implants.
3. Further research should be carried out with long-term clinical trials that evaluate the effect of different tilting angles in all-on four system and zygoma implants on the success rate.

## Data Availability

All data generated or analyzed during this study are included in this published article. Data supporting this research article are available from the corresponding authors on reasonable request.

## Abbreviations

FEA: Finite element analysis
3-D: 3-Dimensional
CT: Computed Tomography
CBCT: Cone Beam Computed Tomography
DICOM: Digital Imaging and Communications in Medicine
stl: Standard Tessellation Language
DOF: Degree of freedom
MPa: Megapascal
N: Newton
